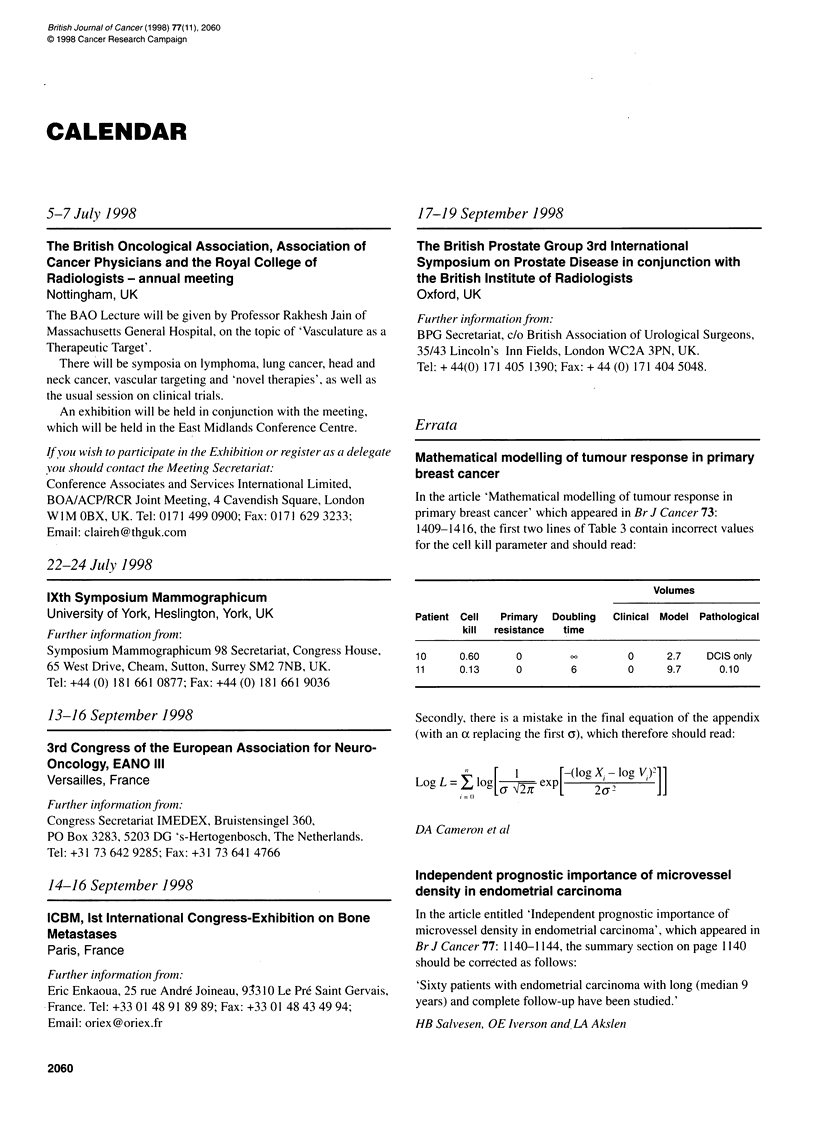# Calendar

**Published:** 1998-06

**Authors:** 


British Journal of Cancer (1998) 77(11), 2060

© 1998 Cancer Research Campaign

### CALENDAR

### *5–7 July 1998*

### The British Oncological Association, Association of Cancer Physicians and the Royal College of Radiologists – annual meeting
Nottingham, UK

The BAO Lecture will be given by Professor Rakhesh Jain of Massachusetts General Hospital, on the topic of 'Vasculature as a Therapeutic Target'.

There will be symposia on lymphoma, lung cancer, head and neck cancer, vascular targeting and 'novel therapies', as well as the usual session on clinical trials.

An exhibition will be held in conjunction with the meeting, which will be held in the East Midlands Conference Centre.

*If you wish to participate in the Exhibition or register as a delegate you should contact the Meeting Secretariat:*
Conference Associates and Services International Limited, BOA/ACP/RCR Joint Meeting, 4 Cavendish Square, London W1M 0BX, UK. Tel: 0171 499 0900; Fax: 0171 629 3233; Email: claireh@thguk.com

### *22–24 July 1998*

### IXth Symposium Mammographicum
University of York, Heslington, York, UK

*Further information from:*
Symposium Mammographicum 98 Secretariat, Congress House, 65 West Drive, Cheam, Sutton, Surrey SM2 7NB, UK.
Tel: +44 (0) 181 661 0877; Fax: +44 (0) 181 661 9036

### *13–16 September 1998*

### 3rd Congress of the European Association for Neuro-Oncology, EANO III
Versailles, France

*Further information from:*
Congress Secretariat IMEDEX, Bruistensingel 360, PO Box 3283, 5203 DG 's-Hertogenbosch, The Netherlands.
Tel: +31 73 642 9285; Fax: +31 73 641 4766

### *14–16 September 1998*

### ICBM, Ist International Congress-Exhibition on Bone Metastases
Paris, France

*Further information from:*
Eric Enkaoua, 25 rue André Joineau, 93310 Le Pré Saint Gervais, France. Tel: +33 01 48 91 89 89; Fax: +33 01 48 43 49 94; Email: oriex@oriex.fr

### *17–19 September 1998*

### The British Prostate Group 3rd International Symposium on Prostate Disease in conjunction with the British Institute of Radiologists
Oxford, UK

*Further information from:*
BPG Secretariat, c/o British Association of Urological Surgeons, 35/43 Lincoln's Inn Fields, London WC2A 3PN, UK.
Tel: + 44(0) 171 405 1390; Fax: + 44 (0) 171 404 5048.